# [Retracted]Oblongifolin C reverses GEM resistance via suppressing autophagy flux in bladder cancer cells

**DOI:** 10.3892/etm.2026.13181

**Published:** 2026-05-11

**Authors:** Zhilong Huang, Tingting Wang, Wenjun Xia, Qing Li, Xinlei Chen, Xiaoli Liu, Peng Wei, Wenping Xu, Meirong Lv

Exp Ther Med 20:1431–1440, 2020; DOI: 10.3892/etm.2020.8856

Following the publication of the above paper, it was drawn to the Editors' attention by a concerned reader that the immunohistochemical image shown in Fig. 1C on p. 1434 and certain of the western blot data featured in Figs. 3B and 4B were strikingly similar to data that had already appeared previously in articles written by different authors at different research institutes.

In view of the fact that the abovementioned data had already apparently been published previously, the Editor of *Experimental and Therapeutic Medicine* has decided that this paper should be retracted from the Journal. The authors were asked for an explanation to account for these concerns, but the Editorial Office did not receive a reply. The Editor apologizes to the readership for any inconvenience caused.

